# Comprehensive Quality Assessment of *Brassica napus* L. Seeds via HPTLC, LC-QToF, and Anatomical Investigation

**DOI:** 10.3390/molecules29132965

**Published:** 2024-06-21

**Authors:** Nazym Tileuberdi, Kumar Katragunta, Sebastian John Adams, Jennyfer A. Aldana-Mejía, Ardak Omarbekova, Bharathi Avula, Ikhlas A. Khan, Aknur Turgumbayeva, Samir A. Ross

**Affiliations:** 1 Higher School of Medicine, Al-Farabi Kazakh National University, Almaty 050040, Kazakhstan; nazym_98@mail.ru (N.T.); turgumbayeva.aknur@med-kaznu.com (A.T.); 2 National Center for Natural Products Research, School of Pharmacy, The University of Mississippi, University, MS 38677, USA; kkatragu@olemiss.edu (K.K.); jasabest@olemiss.edu (S.J.A.); jaaldana@olemiss.edu (J.A.A.-M.); bavula@olemiss.edu (B.A.); ikhan@olemiss.edu (I.A.K.); 3 School of Pharmacy, Asfendiyarov Kazakh National Medical University, Almaty 050012, Kazakhstan; ardashka.0892@mail.ru; 4 Division of Pharmacognosy, Department of Biomolecular Sciences, School of Pharmacy, University of Mississippi, Oxford, MS 38677, USA

**Keywords:** Brassicaceae, histochemistry, flavonoids, microscopy, phytochemistry, quality control

## Abstract

The Brassicaceae family, commonly referred to as cruciferous plants, is globally cultivated and consumed, with the *Brassica* genus being particularly renowned for its functional components. These vegetables are rich sources of nutrients and health-promoting phytochemicals, garnering increased attention in recent years. This study presents a comprehensive microscopic, chromatographic, and spectroscopic characterization of *Brassica napus* L. seeds from Kazakhstan aimed at elucidating their morphological features and chemical composition. Microscopic analysis revealed distinct localization of flavonoids, total lipids, and alkaloids. High-performance thin-layer chromatography (HPTLC) analysis of seed extracts demonstrated a complex chemical profile with significant quantities of non-polar compounds in the hexane extracts. Additionally, methanolic extracts revealed the presence of diverse chemical compounds, including alkaloids, flavonoids, and glucosinolates. The chemical composition exhibited varietal differences across different *Brassica* species, with *B. napus* L. seeds showing higher concentrations of bioactive compounds. Furthermore, liquid chromatography–quadrupole time-of-flight mass spectrometry (LC-QToF-MS) analysis provided insights into the chemical composition, with sinapine isomers, feruloyl, and sinapoyl choline derivatives as major compounds in the seeds. This study contributes to a better understanding of the chemical diversity and quality control methods’ approximations of *B. napus* L. seeds, highlighting their importance in functional food and nutraceutical applications.

## 1. Introduction

The genus *Brassica* L., belonging to the family Brassicaceae, includes crops such as cabbage, turnips, and rapeseed. There are up to 50 types of *Brassica* plants, distributed mainly in the Mediterranean, as well as in America, Central Europe, and Central and East Asia. The second largest oilseed crops belong to the genus *Brassica*. Six species of the genus *Brassica*, namely, *Brassica oleracea* L., *Brassica rapa* L., *Brassica napus* L., *Brassica carinata* A.Braun, *Brassica nigra* (L.) W.D.J.Koch, and *Brassica juncea* (L.) Czern., are widely used as oilseeds, seasonings, fodder, and vegetable crops throughout the world [1]. *Brassica* vegetables are of great economic importance throughout the world, and *Brassica* plants are now grown together with cereals and form the basis of the world’s food supply. The phytochemical screening of *Brassica* plants revealed the presence of some active ingredients, such as alkaloids, cardiac glycosides, coumarins, flavonoids, phenols, phlobatannins, quinines, reducing sugar, resins, saponins, steroids, tannins, terpenoids, xanthoproteins, and volatile oil and carboxylic acid compounds, in varying concentrations [2]. Recently, special attention has been paid to *Brassica* vegetable seeds through phytochemical screening in several studies, which have shown that *Brassica* seeds, like all other organs of the vegetative system, are very rich in nutrients (carbohydrates, vitamins, and minerals) and contain a wide range of different biologically active secondary metabolites with medicinal value (primarily phenolic compounds, glucosinolates, and carotenoids). *Brassica* vegetables are generally rich in polyphenols and contain polyphenolic compounds similar to other members of the genus [3]. The most common and diverse group of polyphenols, *Brassica* spp., are represented by flavonoids (mainly flavonols and anthocyanins) and hydroxycinnamic acids [4]. This wealth of nutritional and medicinal components of *Brassica* seeds has strong biological potential, mainly antioxidant, antiproliferative, antimicrobial, anti-inflammatory, and neuroprotective properties, among others [5]. Variability in oil content is due to genetic differences between *Brassica* species, environmental conditions, and agricultural practices [6]. Among all *Brassica* species, *B. napus* is one of the most important sources of edible oil. According to the USDA, *B. napus* is the second largest oilseed sequence in the world, with a production of 71.94 million tons, and the third largest vegetable oil sequence [5]. Rapeseed (*B. napus*) is an annual herbaceous plant of the genus *Brassica,* containing a wide range of biologically active components. Rapeseed is a natural amphidiploid originated from the crossing of turnip (*B. rapa*) with garden cabbage (*B. oleracea*). *B. napus* is cultivated in large volumes in China, Canada, the USA, India, European countries, Russia, and Kazakhstan. In Kazakhstan, *B. napus* is grown mainly in North and East Kazakhstan, as well as in the Kostanay, Turkistan, and Almaty regions [7]. Rapeseed oil ranks in third place in terms of production, after palm and soybean oils [8]. Rapeseed oil is low in saturated fatty acids (SFAs) but rich in unsaturated fatty acids, including oleic acid, linoleic acid, and alpha-linoleic acid. As reported by previous researchers, the content of unsaturated fatty acids in rapeseed oil can reach 90%, which, however, also varies depending on the species and planting area. In addition, rapeseed oil contains a wide range of fat-soluble microelements, such as polyphenols, tocopherols, phytosterols, carotenoids, chlorophylls, and vitamins [9]. Rapeseed is used in the diet to strengthen vascular walls, reduce cholesterol, increase immune activity, improve mental function and vision, and strengthen muscles and bones.

Macro- and microscopic studies are required for authenticating and identifying genuine botanicals. SEM is a useful tool to determine the characteristics of a plant seed, but also to compare and identify possible variations in seeds from the same species, but from different geographic regions [10]. Light microscopy (LM) and fluorescence microscopy (FM) for detailed histology and histochemistry and scanning electron microscope (SEM) techniques were used to study the surface topography of the seeds in detail. Phytochemical assessment is one of the quality assessment tools, including preliminary phytochemical screening, chemo-profiling, and analysis of marker indicators using modern analytical methods. Over the past two decades, high-performance thin-layer chromatography (HPTLC) has become important for qualitative and quantitative phytochemical analysis of herbal medicines and formulations. This includes TLC fingerprint profiles and estimation of chemical markers. In this article, we conducted a study on *Brassica* seeds ultrasound extracts for assessment of chemical composition by the HPTLC method and its quality investigation by LC-QToF [11].

This study examined the chemical composition and anatomical attributes of *B. napus* seeds cultivated in the Almaty region of Kazakhstan. A comparative analysis was conducted with various cultivars of *B. napus* seeds cultivated in Canada, the United States, and Russia. This study holds significant implications for quality control and chemotaxonomy within the realm of *Brassica* seed production.

## 2. Results

### 2.1. Microscopic Characterization of B. napus Seeds

**Figure 1:**
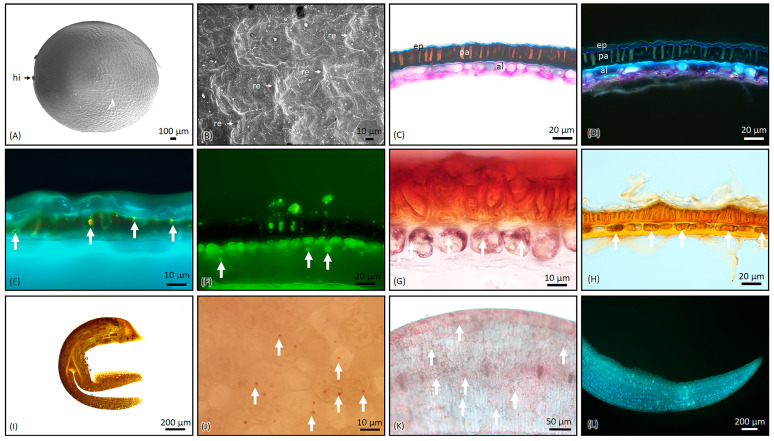
Micro-morphology and histochemistry of seeds of *B. napus*. (**A**,**B**) Scanning electron microscopic observation of seed and close view of the surface texture, respectively. (**C**) Stained with TBO. (**D**) The same section is viewed under UV. (**E**) Stained for flavonoids and viewed under UV (arrows indicate). (**F**) Stained for total lipids with Fluorol Yellow 088 (arrows indicate). (**G**) The section was stained with Nadi reagent for acidic lipids (arrows indicate this). (**H**) Dragendorff reagent stained for alkaloids (arrows indicate). (**I**,**J**) Cotyledon stained with Lugol’s Iodine for starch grains (arrows indicate). (**K**) Cotyledon stained for total lipids with Sudan III (arrows indicate). (**L**) Cotyledon under UV. hi, hilum; re, reticulation; ep, epidermal layer; pa, palisade layer; al, aleurone layer. The cross-section of the seed epidermis has three layers, namely, the outer epidermal layer, palisade, and aleurone cell layer. The outer epidemical layer is a thin, single layer, followed by the palisade layer, which is thick and composed of sclerenchymatous cells. In the third layer are oval to elongated cells filled with aleurone grains (**C**,**D**). Flavonoids were present in the palisade layer (**E**), and the same cells were composed of total lipids (**F**) and yielded a positive reaction to acidic lipids with Nadi stains (**G**). The Dragendorff reagent confirmed the presence of alkaloids (**H**). There was an absence of the endosperm region; thus, this seed belongs to the non-endospermic seed group. The interspace was fully occupied by the cotyledon, which serves as the food storage organ. The cotyledon is conduplicate, filled with very minute starch grains (**I**,**J**) and total lipids (**K**,**L**). No calcium oxalate crystals were present in the seed.

The seeds of *B. napus* have an oval to round shape and external morphology, with a flattened nature or a depression around the hilum. They are black or brown in color, about 0.5–1.0 mm in length, and 0.3–0.8 mm in width. The surface of the seeds have a fine, medium-interspace, stipulated reticulation. The seeds do not have wings or any extra tissue cover and there was an absence of mucilage occurrence on soaking (Figure 1A,B).

**Figure 2:**
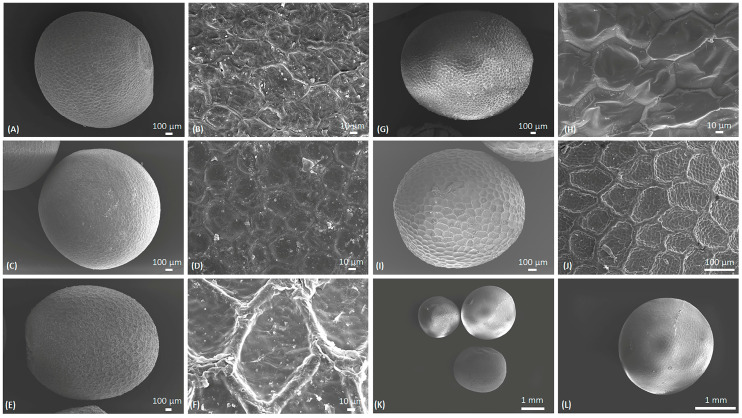
Scanning electron micrograph of the Brassica species. (**A**,**B**) *B. napus* (Red Russian Kale), (**C,D**) *Brassica rapa*, (**E**,**F**) *Brassica juncea*, (**G**,**H**) *Brassica oleracea*, (**I**,**J**) *Brassica nigra*, and (**K**,**L**) *Brassica* mixed sample.

The seeds of different species exhibited similarities in their external morphologies, particularly in their shape. The seeds studied were mostly oval to round in shape. The cultivar of *B. napus* (Red Russian Kale; Figure 2A,B) also showed the same shape and size, although the color varied. However, there were slight variations in other species. For example, *B. rapa* had oval seeds that were often angular, with thick lines of reticulation on the seed surface and small interspaces (Figure 2C,D). Meanwhile, *B. juncea* were round and slightly elongated, with a slightly raised surface. The reticulation showed thick lines of large interspaces (Figure 2E,F). *B. oleracea* (Figure 2G,H) had angular seeds with a prominent radicle and an uneven surface. This characteristic is similar to *B. alba* (synonymous with *B. oleracea*). In *B. nigra* (Figure 2I,J), the surface was oval to round and slightly angular, with a thick reticulation surface. The mixed sample consisted of the seeds of *B. napus*, *B. oleracea*, and *B. nigra* based on the morphological similarities (Figure 2K,L).

### 2.2. HPTLC Analysis of Brassica Seeds’ Extracts

**Figure 3:**
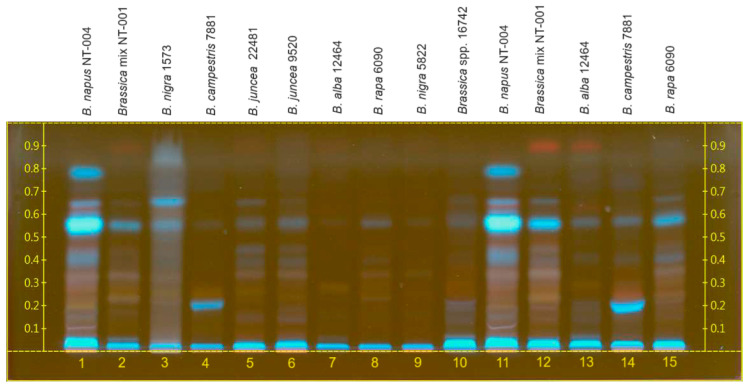
Chromatogram of different Brassica seeds’ hexane extracts. Lanes 11–15 are selected samples at a higher concentration.

A comparative assessment of the qualitative profile of *Brassica* seeds from Kazakhstan was conducted against other seed samples using HPTLC, aiming to identify potential differences in chemical composition. Compared to the other analyzed samples, *B. napus* seeds from Kazakhstan (NT-004 sample) exhibited greater complexity in the chemical profile as well as a higher concentration of secondary metabolites. Most of the biologically active compounds were found in *B. napus* L. (NT-004) hexane seed extract, which in large quantities are non-polar compounds (Figure 3). In the hexane extract of *B. napus*, as a result of examination under UV (254 nm), the main spots were visible at R*_f_* 0.05, 0.4, 0.55, 0.68, and 0.8 (blue). The major compound of *B. napus*, the blue band at R*_f_* 0.55, was also present in lower concentrations in the samples from *B. nigra* (1573), *B. juncea* (22,481 and 9520), *B. alba* (12,464), *B. campestris* (7881), *B. rapa* (6090), and the *Brassica* mix (NT-001).

*B. juncea*’s samples (22,481 and 9520) presented a similar HPTLC profile to *B. napus*. Instead, *B. nigra* presented blue bands with R*_f_* values of 0.55 and 0.65, while *B. campestris* presented a band around 0.22. *B. alba* and *B. rapa* seeds presented the lowest concentrations of the compounds present on other *Brassica* samples.

**Figure 4:**
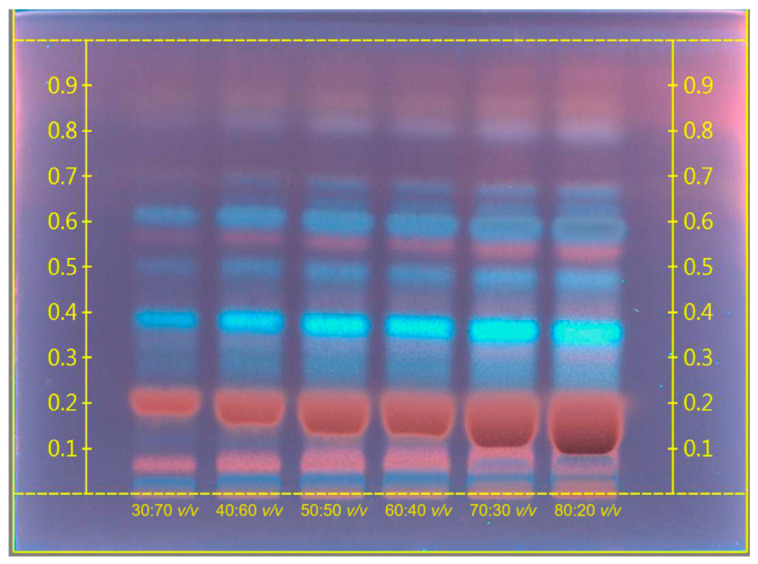
Chromatogram of methanolic *B. napus* seed extract in different concentrations.

For *B. napus* seeds, methanolic extracts were also obtained and analyzed in different concentrations by HPTLC analysis with mobile phase, toluene:ethyl acetate:methanol, at a ratio of 4:10:5 *v*/*v*/*v*. Following the development, the TLC plate was dried on a CAMAG TLC plate heater III at 110 °C for 8 min, and immediately scanned at λ = 254 nm. The chromatogram of methanolic *B. napus* seed extract is presented in Figure 4.

The HPTLC results showed the presence of different chemical compounds in methanolic crude *B. napus* extract. In this extract (254 nm), the main spots were visible at R*_f_* 0.08, 0.2, and 0.58 (red), and 0.4, 0.5, and 0.63 (blue). After derivatization of the plates with iodine, it yielded a yellow color, which demonstrated the major presence of alkaloids in *B. napus* methanolic extract.

### 2.3. LC-QToF Analysis of Brassica Seeds

An LC-MS/MS-based method for the simultaneous identification and quantification of phenolic compounds, their derivatives, and other compounds is described in this study. This analysis was carried out on four different *B. napus* seed varieties grown in Kazakhstan, America, Canada, and Russia using an LC-MS chromatograph.

The chromatographic separation of chemical constituents was achieved by reverse-phase liquid chromatography, followed by high-resolution mass spectrometric (QToF-MS/MS) analysis. Identification of chemical constituents from various samples of *B. napus* seeds resulted in characterization of 39 compounds, which belonged to various classes of chemistry, such as glucosinolates (**1**–**16**), choline derivatives (**17**–**27**), and flavonoids (**28**–**35**), and other types of constituents, such as carbohydrates (**36**), dipeptides (**37**), hydroxy cinnamic acid derivatives (HCA) (**38**), and spermidine amide (**39**). Among the tentatively identified glucosinolates, three different moieties were identified: aliphatic (**1**–**10**), aromatic (**11**–**13**), and indole glucosinolates (**14**–**16**). Four different samples of *B. napus* seeds were analyzed to compare the differences between them. Based on the chromatographic profile and segregation of tentatively identified compounds, there was no substantial difference observed between their chemical profiles. Among all identified compounds, sinapin was the major compound in *B. napus* seeds belonging to choline derivatives. A total of 33 compounds were tentatively identified, which are listed in Table 1 along with their chromatographic retention time, molecular formula, adduct ions observed in positive and negative polarities, their corresponding fragment ions, and possible names of the compounds based on the observed molecular features. Figure 4 represents the LC-MS chromatograms, with the identified compounds labeled based on the compound number.

#### 2.3.1. Glucosinolates (**1**–**16**)

Glucosinolates are *β*-thioglucoside *N*-hydroxysulfates with a side chain, such as aliphatic, aromatic, or indole moieties. The glucosinolates were reported widely in vegetables and herbs, such as *B. napus*. Here in this study, we have identified sixteen different glucosinolates, among which aliphatic side-chain-containing glucosinolates were in higher amount compared to aromatic and indole side chain glucosinolates. Considering the presence of the isothiocyanate group, the ionization of glucosinolates favored negative mode compared to positive polarity. At 2.4 min, compound **1** showed a molecular ion peak at *m*/*z* 436.0421 [M-H]^−^ along with fragment ions, such as *m*/*z* 275.0298 [M-H-Glc]^−^, 96.9500 [HSO_4_]^−^, and 74.9908 [OH-N=C=S]^−^. Based on the identified fragments and other molecular features, compound **1** was identified as glucoraphanin. In the glucosinolates’ mass fragmentation, the sulfate and isothiocyanate groups are considered as the characteristic fragment ions. Based on the information provided in Table 1, compounds **1**–**10** were identified as aliphatic glucosinolates. In continuation, at 4.38 min, compound **11** was showing a molecular ion at 424.0382 [M-H]^−^ along with the characteristic fragment ions of *m*/*z* 241.0014, 96.9599 [HSO_4_]^−^, and 74.9904 [OH-N=C=S]^−^, as described in the aliphatic side-chain-containing glucosinolates. Compound **11** was identified as sinalbin, which belongs to aromatic side chain glucosinolates. Similarly, compounds **14**–**16** were identified as indole side-chain-containing glucosinolates. Chromatographic retention time and mass fragment ions’ information for glucosinolates are listed in Table 1. In addition, Figure 4 describes the TCC chromatogram of *B. napus* seed extract (methanolic) samples, along with labeled chromatographic peaks based on the information provided in Table 1.

#### 2.3.2. Choline Derivatives (**17**–**27**)

This class of metabolites were identified at 13.76, 14.0, and 14.8 min retention times, with a molecular peak of *m*/*z* 310.1654 [M]^+^ along with fragment ions of *m*/*z* 251.0906 [M-NH(CH_3_)]^+^, 207.0637 [M-NH(CH_3_)-C_2_H_4_O]^+^, 175.0382 [M-NH(CH_3_)-C_2_H_4_O-CH_3_OH]^+^, 147.0433 [M-NH(CH_3_)-C_2_H_4_O-CH_3_OH-CO]^+^, 119.0484 [M-NH(CH_3_)-C_2_H_4_O-CH_3_OH-2CO]^+^, and 91.0536 [M-NH(CH_3_)-C_2_H_4_O-CH_3_OH-3CO]^+^. Based on the observed fragment ions, the chromatographic peaks were related to sinapine and corresponding isomers. In general, the choline derivatives possessed a characteristic loss of trimethyl amine fragment, ethoxy fragment/s, and consecutive loss of -CO fragments. Based on the observed fragments listed in Table 1, **17**–**27** were tentatively identified as choline derivatives. A detailed list of fragment ions and their corresponding molecular ions and retention times are provided in Table 1.

#### 2.3.3. Flavonoids (**28**–**35**)

**Figure 5:**
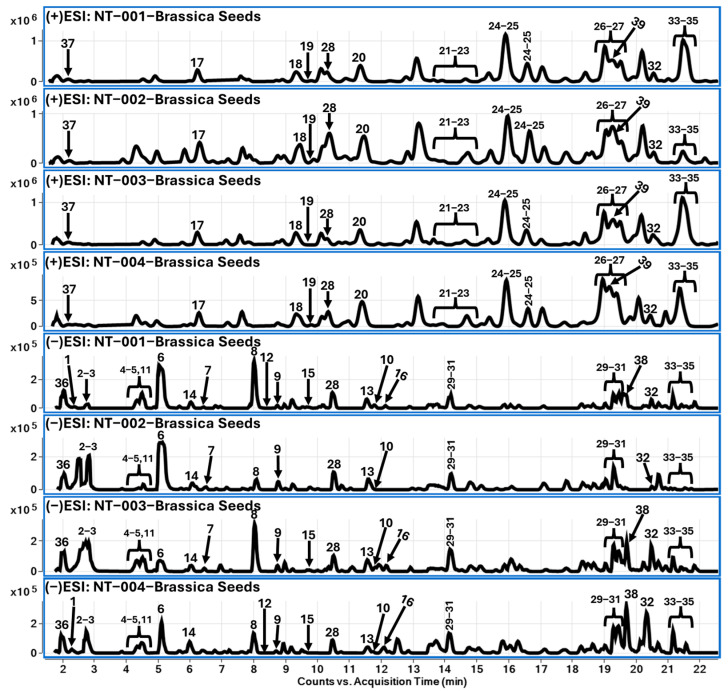
TCC chromatograms (positive and negative ion mode) for *B. napus* seed (methanolic) extracts.

Chromatographic peaks were observed at 10.4, 14.1, 19.0, and 19.6 min, showing *m*/*z* 285.0389 [Kaempferol-H]^−^ fragment ions, and based on the observed molecular ions, these peaks were annotated as kaempferol-sophoroside (**28**) and kaempferol-(sinapoylglucoside)-sophoroside (**29**–**31**). Similarly, compounds **32**–**35** were identified as disinapoyl (*m*/*z* 753.2243 [M-H]^−^ and 959.2819 [M-H]^−^)and trisinapoyl-gentiobiose [18]. In addition, compounds 29–31 were not detected in NT-001 and NT-002 samples, whereas NT-003 contained traceable amounts and NT-004 showed a higher EIC peak intensity compared to the NT-003 sample. Molecular features, such as retention times and adduct ions in positive and negative mode, along with the corresponding fragment ions, are listed in Table 1 and annotated in Figure 5.

#### 2.3.4. Other Compounds (**36**–**39**)

Based on the LC-QToF data, *B. napus* seeds showed minimal amounts of sucrose (**36**), glutamyl-methionine sulfoxide (**37**), sinapoyl malic acid (**38**), and spermidine conjugate (**39**). The chromatographic peak at 2.0 min showed *m*/*z* 341.1089 [M-H]^−^ along with fragment ions, listed in Table 1 as sucrose (**36**). Similarly, at 2.1 min, a molecular ion peak of *m*/*z* 295.0957 [M+H]^+^ was tentatively identified as glutamyl-methionine sulfoxide. Similarly, at 19.68 min, there was a molecular ion peak at *m*/*z* 339.0726 [M-H]^−^ and corresponding fragment ions at *m*/*z* 223.0610 [Sinapoyl-H]^−^ and 149.0239. Based on the observed molecular features, the compound **38** was identified as sinapoyl malic acid, which belongs to hydroxycinnamic acid derivatives. Compound **39** showed *m*/*z* 496.2437 [M+H]^+^ and 530.2081 [M+Cl]^−^, and based on the previously reported studies on *B. napus*, it was identified as spermidine amide (**39**).

## 3. Discussion

Brassica napus seeds are rich in oil content. There are several cultivars and hybrid species of B. napus and other closely related species, such as B. carinata, B. juncea, B. nigra, B. oleracea, and B. rapa, which are used for their oil content [19,20]. According to existing literature, the seed coat of B. napus comprises three discernible cell layers: a palisade layer, multiple layers of crushed parenchyma cells, and a single layer of aleurone cells [21]. Pigment deposition, primarily flavonoids, occurs in both the palisade and crushed layers of the seed parenchyma [22]. According to the literature, the palisade layer is characterized by a higher fiber content [23]. Flavonoids play a pivotal role in the pigmentation process of Brassica seed coats [24], with various groups identified, including flavonols, anthocyanins, phlobaphenes, isoflavones, and proanthocyanidins [25]. Proanthocyanidin, a condensed tannin, is exclusively deposited in the seed coat, contributing significantly to its pigmentation [23,26]. Seed coats of black/brown-seeded Brassica genotypes exhibit higher fiber content and lower protein content compared to yellow-seeded genotypes [23].

In our investigation, seeds of B. napus exhibited positive reactions not only to flavonoids but also to alkaloids, glycosides, lipids, starch, and polysaccharides. Lipids and proteins were segregated into distinct cellular organelles distributed across the aleurone layer of the endosperm and embryonic cells. Polysaccharides in B. napus were predominantly in the form of structural carbohydrates, contributing to the complex composition of the seed coat, which also comprises mucilage and lignin. While carbohydrates are primarily concentrated in the embryo, protein bodies are found in both the aleurone layer and most cells of the cotyledons and radicle. Scanning electron microscopy revealed storage lipids in small, discrete, densely packed droplets surrounding protein bodies and cell nuclei.

The chemical analyses, employing HPTLC and LC-QToF methodologies, unveiled notable disparities in chemical profiles among various Brassica species, with B. napus demonstrating elevated concentrations of bioactive compounds, such as phenols, flavonoids, and glucosinolates. The compositional profile of Brassica seeds varied depending on the cultivar, solvent utilized for extraction, and methods employed for detection or quantification [5]. Different classes of secondary metabolites were present on the Brassica seeds.

Choline derivatives emerged as the major class of compounds present in B. napus seeds, a trend consistent with previous studies [5,27]. Species such as *B. campestris*, *B. napus*, and *B. rapa* exhibited high concentrations of these secondary metabolites in seed meals [5]. Our results indicated that samples from Kazakhstan share similarities with other Brassica samples, particularly regarding the prevalence of choline derivatives, especially sinapine isomers. LC-MS analysis of B. napus seeds from Korea also identified sinapine as a major compound [28].

LC-MS analysis revealed significant varietal differences in both total and individual glucosinolate content, contrasting with previous studies on Japanese rapeseed varieties [29]. Predominantly, aliphatic glucosinolates, such as feruloyl choline furuyl ester and sinapoyl choline feruloyl ester derivatives, were observed across all analyzed samples. Progoitrin and gluconapin, both glucosinolates, serve as chemical markers of *B. napus* seeds [5], with our results indicating their major presence in the analyzed samples (**2**, **6**), particularly in Red Russian Kale cultivar seeds. Interestingly, while glucoiberverin and sinalbin were present across all samples analyzed, they were notably absent in the B. napus collected from Kazakhstan.

Brassica seeds exhibited a notable richness in polyphenols, including hydroxycinnamic acids (caffeic, ferulic, sinapic, and p-coumaric acids or their derivatives) and flavonoids (such as quercetin, kaempferol, and isorhamnetin derivatives) [5,30]. Hydroxycinnamic acids have been identified in select species of B. juncea, B. rapa, and B. campestris [5]. Previous studies have highlighted B. juncea seeds as rich reservoirs of polyphenols, including vanillin, catechin, and quercetin [31]. However, compared to glucosinolates and choline derivatives, Brassica seeds analyzed in this study contained minimal amounts of flavonoids, primarily comprising a sinapoyl moiety backbone attached to flavonoids aglycone and gentiobiose. Previous LC-ESI-MS analysis confirmed the presence of kaempferol-3-O-β-sophoroside and 1,2-Di-O-sinapoylgentiobiose in Korean samples [28].

Secondary metabolites, such as carotenoids, saponins, and tannins, were detected in seeds of B. nigra and B. juncea [32,33,34], but were notably not detecable in the Kazakhstan sample. These outcomes offer valuable insights into both chemotaxonomy and quality control within the Brassica genus. By elucidating the chemical composition of Brassica seeds and identifying specific metabolites, such as choline derivatives, flavonoids, and glucosinolates, this research provides essential chemotaxonomic markers for distinguishing between Brassica species. Furthermore, the observed variations in chemical profiles among different varieties highlight the necessity of stringent quality control measures in Brassica seed production and processing, simultaneously promoting their utilization as functional food ingredients and therapeutic agents.

## 4. Material and Methods

### 4.1. Botanical Materials

In this study, seeds of *Brassica napus* (sample code—NT-004) were collected at the Kazakh Research Institute of Agriculture and Plant Growing in Almaty during the seed ripening period. For comparison purposes, we also studied the other cultivars of *B. napus* seeds: Red Russian Kale (NT-002) and *Brassica napus* subsp. *napus* (NT-003), and other species of *Brassica* plant materials were assigned unique NCNPR numbers: *B. rapa* (6090), *B. oleracea* (16,742), *B. nigra* (1573), *B. juncea* (22,481, 9520), *B. alba* (12,464), synonym of *B. oleracea* L., *B. campestris* (7881), synonym of *B. rapa*, and a mix of different *Brassica* spp. (NT-001), deposited at the Botanical Repository of the National Center for Natural Products Research, University of Mississippi, University, MS, USA.

### 4.2. Sample Preparation for Microscopy

Cross-sections of seeds for detailed anatomical studies were carried out using hand sections (~50 µm-thick) and microtome sections (15 µm-thick), and sections were stained with Toluidine Blue O (TBO) for basic histology observation. Histochemical evaluations of transverse sections were performed using stains specifically for localizing various substances, such as starch, lignin, terpenes, flavonoids, total lipids, and alkaloids. Sudan III and Fluorol Yellow 088 were used for total lipids and some essential oils [35,36], Dragendorff reagent was used for alkaloids and acidic phospholipids [37,38], Auramine O was used for lignin and cutin [39,40,41], and Naturstoff reagent A (diphenylboric acid 2-aminoethyl ester (DPBA)) was used for confirmation of flavonoids under UV fluorescence [42,43]. All mounts were prepared on glass slides with water. Photomicrographs were obtained using an Olympus BX53 compound microscope equipped with an Olympus DP74 camera system with fluorescence imaging. Images were processed using Olympus CellSens standard 2 (version 3.1, build 21,199) imaging software (Olympus Corp., Tokyo, Japan). Micrometrics of leaf anatomical characteristics were measured and statistical mean values were consolidated.

### 4.3. Sample Preparation for Scanning Electron Microscopy (SEM)

Specimens fixed in formaldehyde alcohol acetic acid (FAA) were washed with water and passed through 30%, 50%, 70%, 90%, and 100% ethanol solutions. The samples were dried using a Leica CPD300 critical point dryer (Leica Microsystems, Wetzlar, Germany) supplied with liquid CO_2_. Dried samples were mounted on aluminum stubs with double-sided adhesive carbon tape then coated with platinum using a Desk V HP sputter coater (Denton Vacuum, Moorestown, NJ, USA) supplied with argon gas. The samples were imaged using a JSM-7200FLV field-emission SEM (JEOL Ltd., Tokyo, Japan).

### 4.4. Sample Preparation for HPTLC Analysis

The method was developed using a CAMAG HPTLC system (CAMAG, Muttenz, Switzerland) on Brassica seed hexane extracts and rapeseed (B. napus) methanolic extracts. The analysis was carried out on nano-silica HPTLC plates w/UV254, 10 × 20 cm, and 200 µm layer thickness. The mobile phase for hexane Brassica seed extract was hexane:ethyl acetate at a ratio of 10:1.5 *v*/*v*, and for methanolic rapeseed extract was toluene:ethyl acetate:methanol at a ratio of 4:10:5 *v*/*v*/*v*.

The development of the plates and the drying process were carried out on the CAMAG Automatic Developing Chamber 2 (ADC2). Each extract was applied by spraying using a CAMAG HPTLC system. Detection and documentation were processed using the CAMAG TLC Visualizer 2 under UV light of 366 nm. For the collection of the data analysis, CAMAG HPTLC Software vision CATS (version 3.2 SP2) was used (CAMAG, Muttenz, Switzerland). The application type was bands and the time for development was 15 min.

Extraction was carried out on different Brassica seeds in an ultrasonic bath with a temperature of 60 °C for 2 h by using hexane and methanol solvents to compare the extraction efficiency at a ratio of 1:2 *w*/*v*. The extracts were filtered with Whatman filter paper and residual solvents were removed using a rotary evaporator.

### 4.5. LC-QToF Sample Preparation

About 1 g of dried samples of B. napus seeds (fine powdered) were weighed and subjected to ultra-sonication for 30 min using 4 mL of methanol solvent, individually. Further, these samples were centrifuged at 4700 rpm for 15 min. The supernatant solution was removed from seed samples and transferred into a volumetric flask of 10 mL. A similar procedure of ultrasonication and centrifugation was repeated two more times, using 3 mL of methanol each time. After a third extraction followed by centrifugation, the volume of supernatant solution in the volumetric flask was adjusted to 10 mL using methanol as a diluent. Further, these sample solutions were filtered using 0.45 µ PTFE filters before being subjected to LC-QToF-MS analysis.

#### Instrumentation and Analytical Conditions

The 1290 UHPLC system (Agilent Technologies, Santa Clara, CA, USA) consists of a binary pump, and the chromatographic separation was achieved on a Poroshell EC-C18 column (2.1 × 150 mm, 2.7 µm). Mobile phase contained 0.1% formic acid in water (A) and 0.1% formic acid in acetonitrile (B). Chromatographic separation involved a 0.23 mL/min flow rate with, initially, 1%B to 5%B (0–4 min), 5%B to 10%B (4–7 min), 10%B to 13%B (10–15 min), 13%B to 40%B (15–20 min), and 40%B to 70%B (20–25 min), followed by a 3 min wash with 100%B and equilibrated for 5 min with initial gradient 99%A/1%B. Column temperature was maintained at 45 °C. A 1 microliter sample was injected onto the column to separate the compounds and further screened at 330 nm in a DAD detector.

In continuation of the UHPLC-DAD analysis, the chromatographic system was combined with the QToF-MS system (G6545B, Agilent Technologies, Santa Clara, CA, USA) with an electro-spray ionization (ESI) source, which facilitated the ionization of secondary metabolites in the medium, polar-to-polar in nature. To ensure the ionization of compounds, the analysis was performed in both positive and negative polarities. For QToF-MS analysis, the following parameters were applied in the low-vacuum region: dry gas temperature at 325 °C, dry gas (nitrogen) follow at 13 L/min, sheath gas temperature at 300 °C, sheath gas (nitrogen) at 12 L/min, and nebulizer pressure at 20 psig. In addition, the scan source parameters were adjusted as follows: capillary voltage of 3.0 kV, nozzle voltage at 0 V, and fragmentor voltage at 150 V/175 V, for both positive and negative polarities, respectively. The acquisition range was maintained at *m*/*z* 50–1700 (dynamic range). Nitrogen was used as collision gas for MS/MS experiments and a 45 eV collision energy was applied for the auto-MS/MS fragmentation study. Acquisition and post-run analysis of data were controlled by the MassHunter Qualitative Analysis software platform ver. B.07.00 (Agilent Technologies, Santa Clara, CA, USA). Each sample was analyzed in positive and negative modes over the range of *m*/*z* 50–1700 and an extended dynamic range. Accurate mass measurements were obtained employing ion correction techniques using reference masses at *m*/*z* 121.0509 (protonated purine) and 922.0098 (protonated hexakis [1*H*,1*H*,3*H*-tetrafluoropropoxy] phosphazine or HP-921) in positive ion mode, while *m*/*z* 112.9856 (deprotonated trifluoroacetic acid-TFA) and 1033.9881 (TFA adducted HP-921) were used in negative ion mode.

## 5. Conclusions

The microscopic characterization of *B. napus* seeds revealed a complex structure composed of distinct cell layers in the seed coat, with flavonoids predominantly present in the palisade layer, as well as lipids and alkaloids. These results emphasized that the secondary metabolites are mostly located in the seed coat and the endosperm regions composed of high amounts of fixed oil contents. HPTLC analysis of *Brassica* seed extracts showed significant variation in the chemical profiles among different species, with *B. napus* exhibiting higher concentrations of biologically active compounds. LC-QToF analysis further elucidated the chemical composition of *B. napus* seeds, identifying a diverse range of compounds, including glucosinolates, choline derivatives, and flavonoids, and confirming the prevalence of alkaloidal amines, such as sinapine derivatives. These findings underscore the potential health benefits and nutritional value associated with *Brassica* seeds, highlighting their importance in agricultural and pharmaceutical industries through the development of quality control methods. Further research into the specific bioactive properties of these compounds could contribute to the development of novel therapeutic applications and dietary supplements.

## Figures and Tables

**Table 1:** LC-QToF-MS data for compounds from various seed extracts of *B. napus* L. species.

#	RT (min)	Compound Name [Ref]	Mol. Formula	Exact Mass [M]	[M+H]^+^	Fragment Ions (Positive Mode)	[M-H]^−^	Fragment Ions (Negative Mode)	Samples			
									NT-001	NT-002	NT-003	NT-004
Aliphatic glucosinolates [12]												
1	2.40	Glucoraphanin	C_12_H_23_NO_10_S_3_	437.0484	-	-	436.0421 (436.0411) *	275.0298 [M-H-Glc]^−^, 96.9590 [HSO_4_]^−^, 74.9908 [OH-N=C=S]^−^	+	+	+	+
2	2.80	Progoitrin	C_11_H_19_NO_10_S_2_	389.0450	-	-	388.0383 (388.0378)	259.0155, 241.0024, 135.9690, 96.9600 [HSO_4_]^−^, 74.9910 [OH-N=C=S]^−^	+	+	+	+
3	2.89	Sinigrin	C_10_H_17_NO_9_S_2_	359.0345	-	-	358.0277 (358.0272)	259.0125, 241.0029, 96.9599 [HSO_4_]^−^, 74.9910 [OH-N=C=S]^−^	+	+	+	+
4	4.36	Glucoallysin	C_13_H_25_NO_10_S_3_	451.0641	-	-	450.0572 (450.0568)	259.0132, 112.9854, 96.9600 [HSO_4_]^−^	+	+	+	+
5	4.52	Gluconapoleiferin	C_12_H_21_NO_10_S_2_	403.0607	-	-	402.0540 (402.0534)	259.0116, 96.9601 [HSO_4_]^−^, 74.9909 [OH-N=C=S]^−^	+	+	+	+
6	5.23	Gluconapin	C_11_H_19_NO_9_S_2_	373.0501	-	-	372.0428 (372.0425)	274.9895, 259.0135, 241.0033, 130.0329, 96.9600 [HSO_4_]^−^, 74.9912 [OH-N=C=S]^−^	+	+	+	+
7	6.50	Glucoiberverin	C_11_H_20_NO_9_S_3_^−^	406.0300 [M]^−^	-	-	406.0309 (406.0300)	96.9608 [HSO_4_]^−^, 74.9918 [OH-N=C=S]^−^	+	+	+	ND
8	8.06	Glucobrassicanapin	C_12_H_21_NO_9_S_2_	387.0658	-	-	386.0594 (386.0585)	274.9950, 259.0120, 144.0484, 96.9604 [HSO_4_]^−^, 74.9912 [OH-N=C=S]^−^	+	+	+	+
9	8.78	Glucoerucin	C_12_H_23_NO_9_S_3_	421.0535	-	-	420.0459 (420.0462)	96.9597 [HSO_4_]^−^, 74.9916 [OH-N=C=S]^−^	+	+	+	+
10	11.80	Glucoberteroin	C_13_H_24_NO_9_S_3_^−^	434.0613 [M]^−^	-	-	434.0609 (434.0613)	96.9598 [HSO_4_]^−^, 74.9911 [OH-N=C=S]^−^	+	+	+	+
Aromatic glucosinolates [12]												
11	4.38	Sinalbin	C_14_H_19_NO_10_S_2_	425.0450	-	-	424.0382 (424.0378)	241.0014, 96.9599 [HSO_4_]^−^, 74.9904 [OH-N=C=S]^−^	+	+	+	tr
12	8.40	Glucotropaeolin	C_14_H_19_NO_9_S_2_	409.0501	-	-	408.0430 (408.0428)	96.9593 [HSO_4_]^−^, 74.9901 [OH-N=C=S]^−^	+	ND	ND	+
13	11.54	Gluconasturtiin	C_15_H_21_NO_9_S_2_	423.0658	-	-	422.0589 (422.0585)	259.0113, 96.9599 [HSO_4_]^−^, 74.9913 [OH-N=C=S]^−^	+	+	+	+
Indole glucosinolates [12]												
14	6.05	4-Hydroxy-glucobrassicin	C_16_H_20_N_2_O_10_S_2_	464.0559	-	-	463.0496 (463.0487)	285.0190, 259.0133, 221.0360, 96.9601 [HSO_4_]^−^, 74.9908 [OH-N=C=S]^−^	+	+	+	+
15	9.65	Glucobrassicin	C_16_H_20_N_2_O_9_S_2_	448.0610	-	-	447.0546 (447.0537)	96.9606 [HSO_4_]^−^, 74.9910 [OH-N=C=S]^−^	+	tr	+	+
16	12.10	4-Methoxy-glucobrassicin/Neoglucobrassicin	C_17_H_22_N_2_O_10_S_2_	478.0716	-	-	477.0657 (477.0643)	96.9607 [HSO_4_]^−^, 74.9919 [OH-N=C=S]^−^	+	tr	+	+
Choline derivatives [13]												
17	6.28	Vanilloyl-choline-hexoside	C_19_H_29_NO_9_	415.1842	416.1914 (416.1915) * [M]^+^	223.1436, 194.1166, 138.0908, 118.0860	-	-	+	+	+	+
18	9.34	Sinapoylcholine-hexoside	C_22_H_34_NO_10_^+^	472.2177	472.2179 (472.2177) [M]^+^	261.1310, 251.0914, 239.1489, 207.0651, 175.0387	-	-	+	+	+	+
19	9.59								tr	tr	tr	tr
20	11.30	Benzoylcholine	C_12_H_18_NO_2_^+^	208.1338	208.1335 [M]^+^	149.0590 [M-NH(CH_3_)]^+^, 105.0330 [M-NH(CH_3_)-C_2_H_4_O]^+^, 77.00385 [M-NH(CH_3_)-C_2_H_4_O-CO]^+^	-	-	+	+	+	+
21	13.76	Sinapine/sinapine isomers [14]	C_16_H_24_NO_5_^+^	310.1654	310.1654 [M]^+^	251.0906 [M-NH(CH_3_)]^+^, 207.0637 [M-NH(CH_3_)-C_2_H_4_O]^+^, 175.0382 [M-NH(CH_3_)-C_2_H_4_O-CH_3_OH]^+^, 147.0433 [M-NH(CH_3_)-C_2_H_4_O-CH_3_OH-CO]^+^, 119.0484 [M-NH(CH_3_)-C_2_H_4_O-CH_3_OH-2CO]^+^, 91.0536 [M-NH(CH_3_)-C_2_H_4_O-CH_3_OH-3CO]^+^	-	-	++	++	++	++
22	14.00								+	+	+	+
23	14.80								+	+	+	+
24	15.95	Feruloyl choline furulyl ester	C_25_H_34_NO_8_^+^	476.2279	476.2276 (476.2279) [M]^+^	310.1641, 221.0803, 177.0541, 145.0279	-	-	+	+	+	+
25	16.62								+	+	+	+
26	18.94	Sinapoyl choline feruloyl ester	C_26_H_36_NO_9_^+^	506.2390	506.2379 (506.2390) [M]^+^	251.0926, 207.0657, 175.0396, 147.0447, 104.1069	-	-	+	+	+	+
27	19.48								+	+	+	+
Flavonoids [15,16]												
28	10.40	Kaempferol-sophoroside-glucoside	C_33_H_40_O_21_	772.2062	795.1941 (795.1954) [M+Na]^+^	611.1568 [M+H+Glc]^+^, 449.1059 [M+H+2Glc]^+^, 287.0530 [M+H+3Glc]^+^	771.1988 (771.1989)	609.1471 [M-H-Glc]^−^, 447.0901 [M-H-2Glc]^−^, 285.0389 [M-H-3Glc]^−^	+	+	+	+
29	14.14	Kaempferol-(sinapoylglucoside)-sophoroside	C_44_H_50_O_25_	978.2641	1001.2539 (1001.2533) [M+Na]^+^	611.1604, 287.0558	977.2560 (977.2568)	815.2058, 609.1506, 465.0731, 307.0863	+	+	+	+
30	19.03		C_44_H_50_O_25_	978.2641				815.2058, 653.1535, 447.0937, 285.0382	+	+	+	+
31	19.57		C_44_H_50_O_25_	978.2641				815.2087, 609.1491, 545.0942, 285.0412	+	tr	+	+
32	20.34	Disinapoyl-gentiobiose	C_34_H_42_O_19_	754.2320	777.2216 (777.2213) [M+Na]^+^	553.1532, 411.1420, 207.0648	753.2243 (753.2248)	547.1661, 205.0504	+	+	+	+
33	21.20	Trisinapoyl-gentiobiose	C_45_H_52_O_23_	960.2899	983.2781 (983.2792) [M+Na]^+^	759.2098 [M+Na-Sinapic acid]^+^, 631.1443, 615.1676, 369.1187, 207.0654, 175.0392	959.2819 (959.2827)	591.1734, 427.1225, 247.0612, 223.0620, 205.0509	+	+	+	+
34	21.60								ND	ND	tr	+
35	21.85								+		+	tr
Others												
36	2.00	Sucrose (Carbohydrate)	C_12_H_22_O_11_	342.1162	-	-	341.1089 (341.1095)	179.0557, 161.0457, 119.0345, 89.0242, 59.0139	+	+	+	+
37	2.10	Glutamyl-methionine sulfoxide (Dipeptide)	C_10_H_18_N_2_O_6_S	294.0886	295.0957 (295.0958)	133.0427, 104.1068	-	-	+	+	+	+
38	19.68	Sinapoyl malic acid (HCA) [17]	C_15_H_16_O_9_	340.0794	-	-	339.0726 (339.0722)	223.0610, 149.0239	+	+	+	+
39	19.32	Cyclic (diferulic acid/spermidine) conjugate (Spermidine amide) [16]	C_27_H_33_N_3_O_6_	495.2369	496.2437 (496.2442)	328.2470, 175.0382	494.2303 (494.2297) 530.2081 (530.2063) [M+Cl]-	-	+	+	+	+

* Theoretically accurate mass; ‘+’ indicates presence of compound; ‘ND’ indicates not detected; ‘++’ indicates strong intensity; ‘tr’ indicates presence of compound in trace amount (intensity < 1000 cps). NT-001: *Brassica* mixed seeds; NT-002: Red Russian Kale seeds; NT-003: *Brassica napus* seeds Canola; NT-004: *Brassica napus* seeds Kazakhstan.

## Data Availability

Data are contained within the article.
